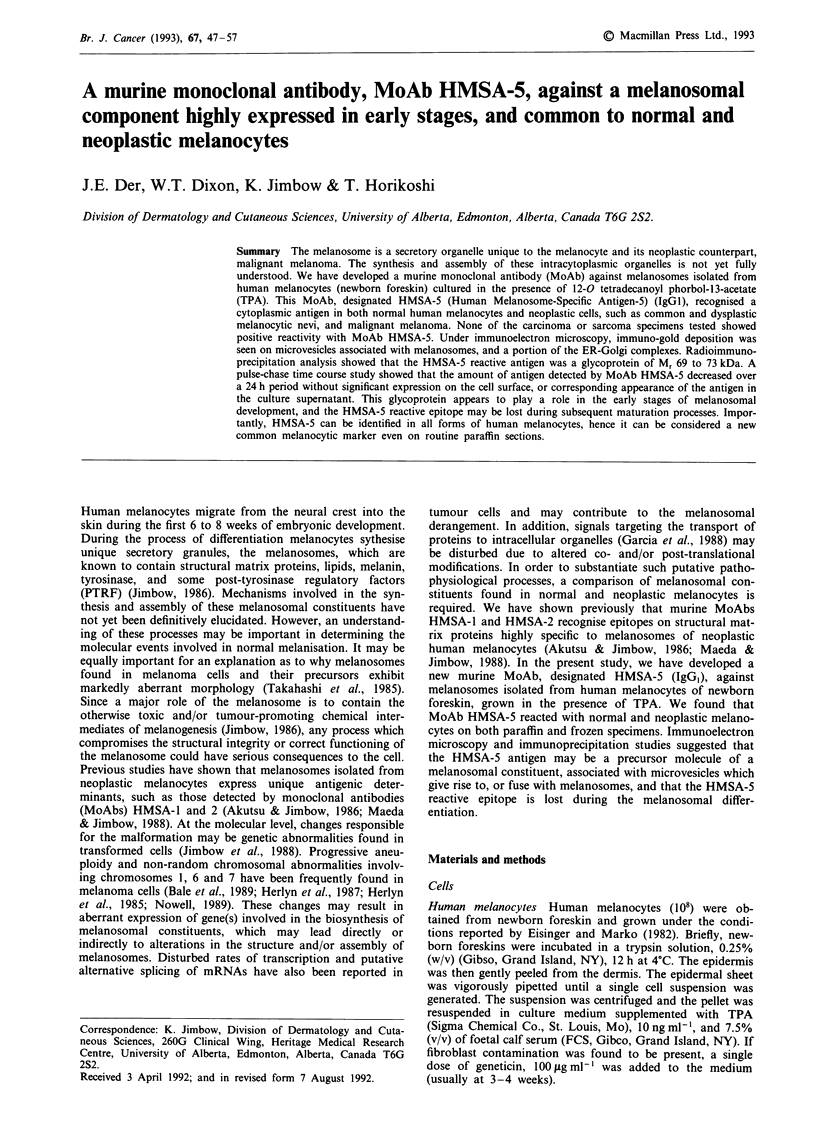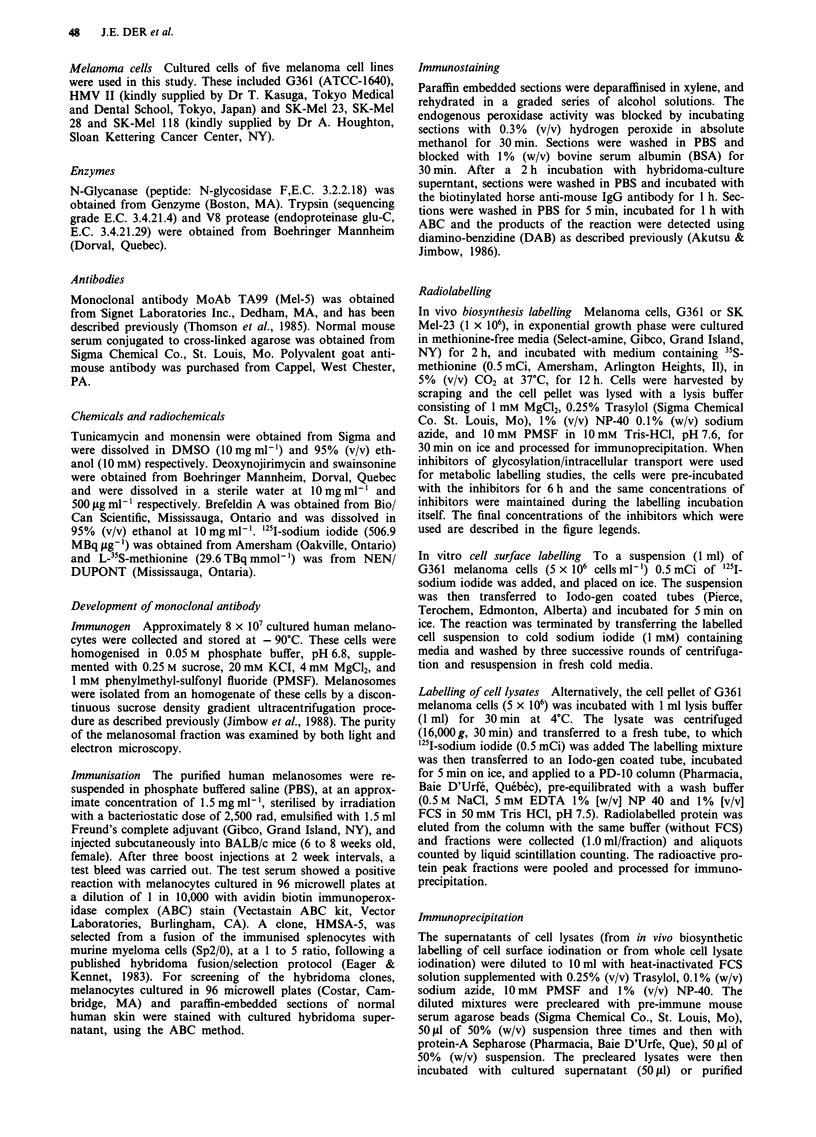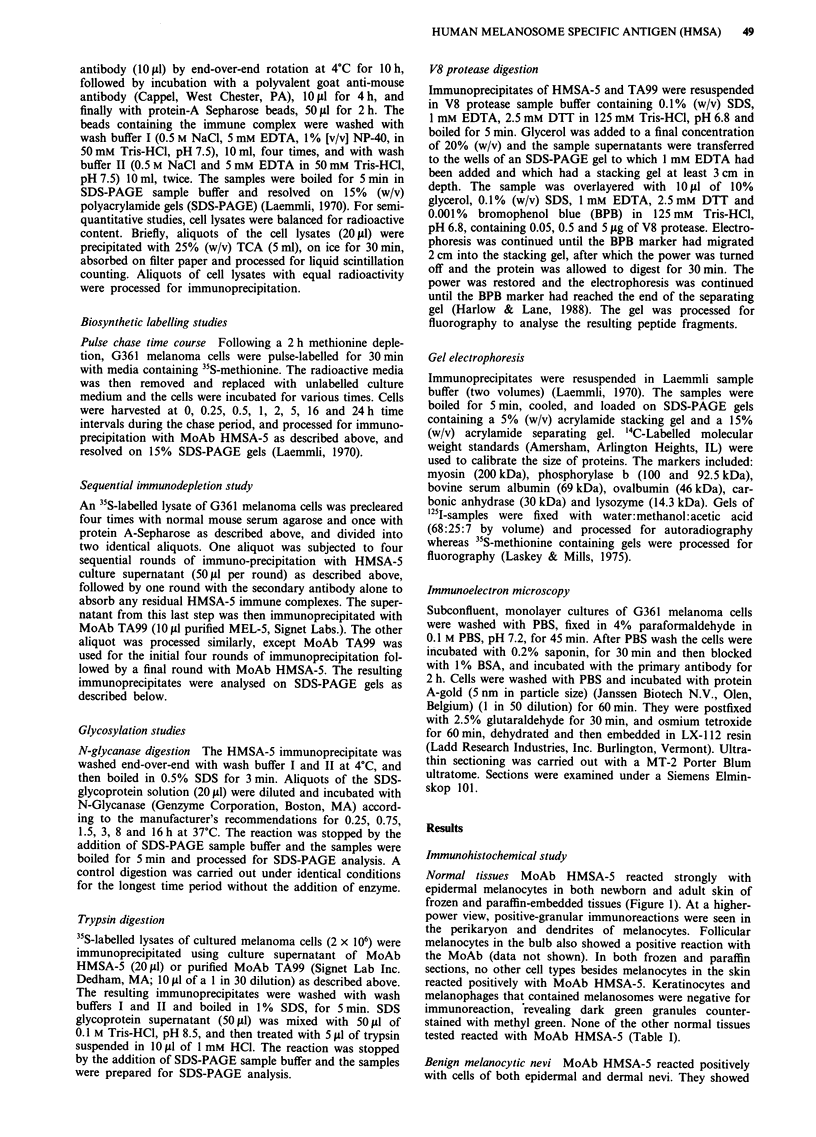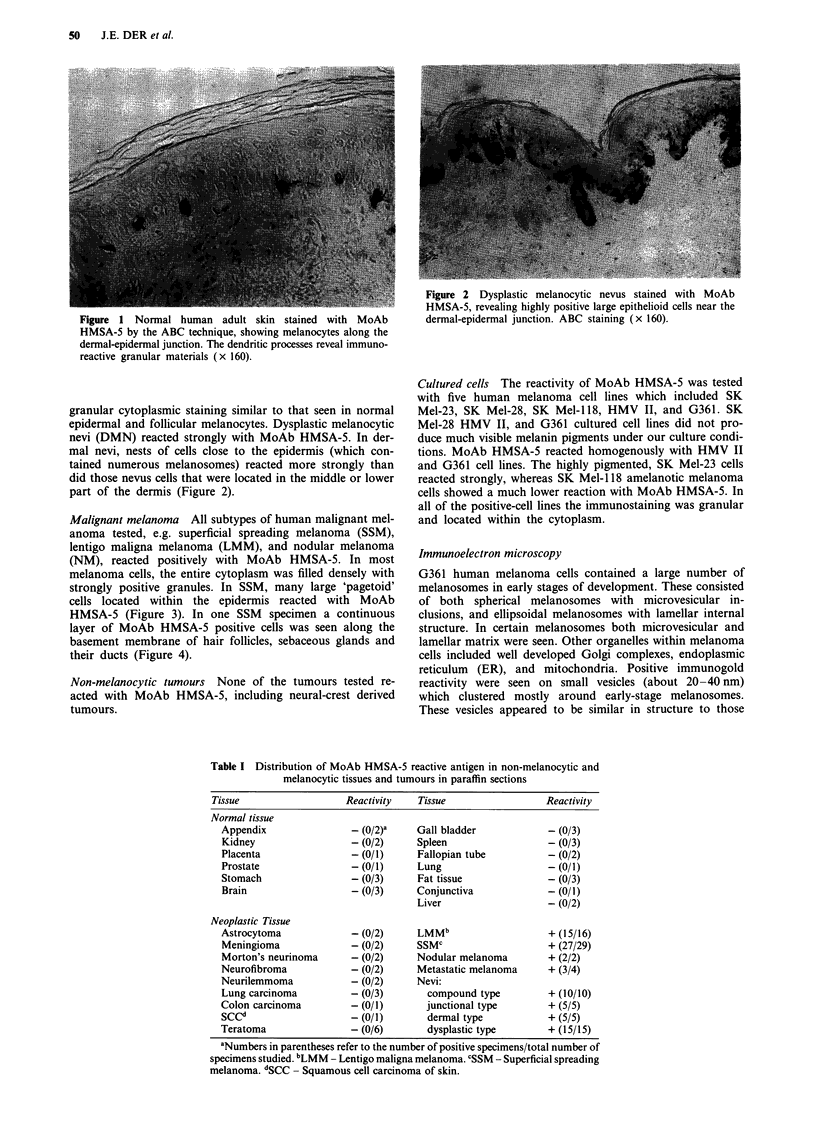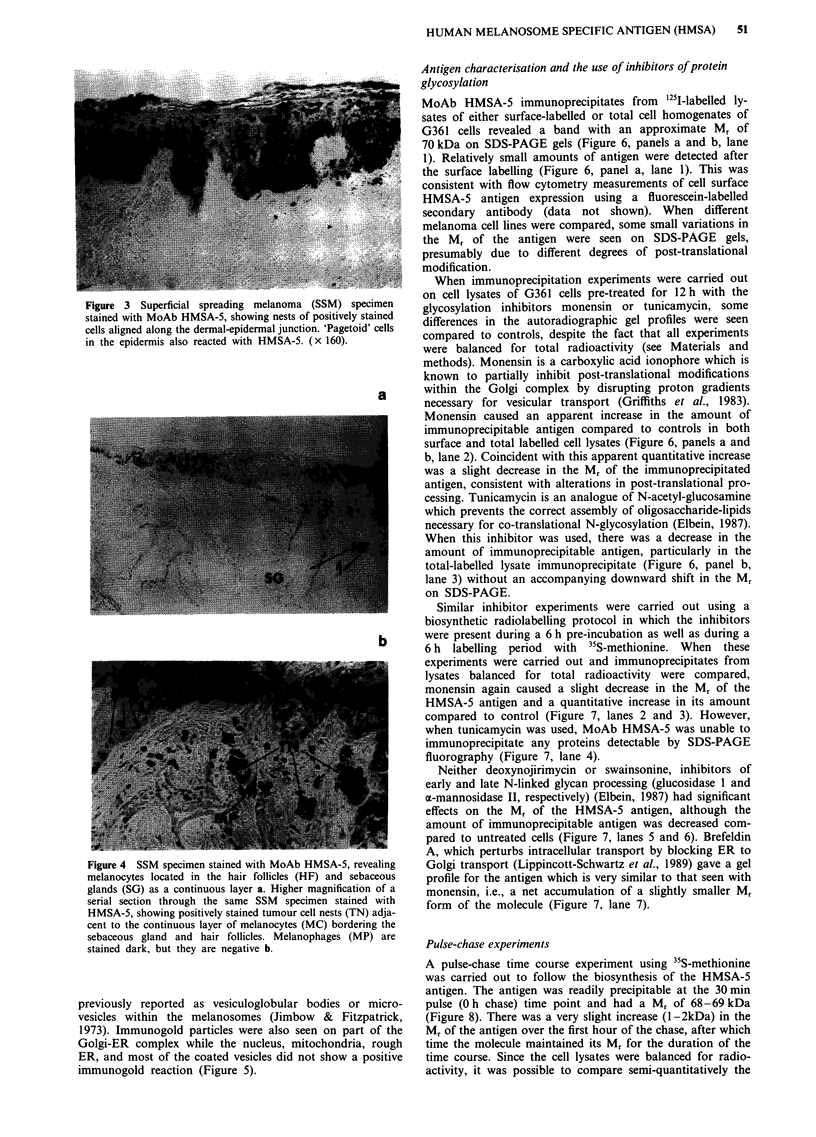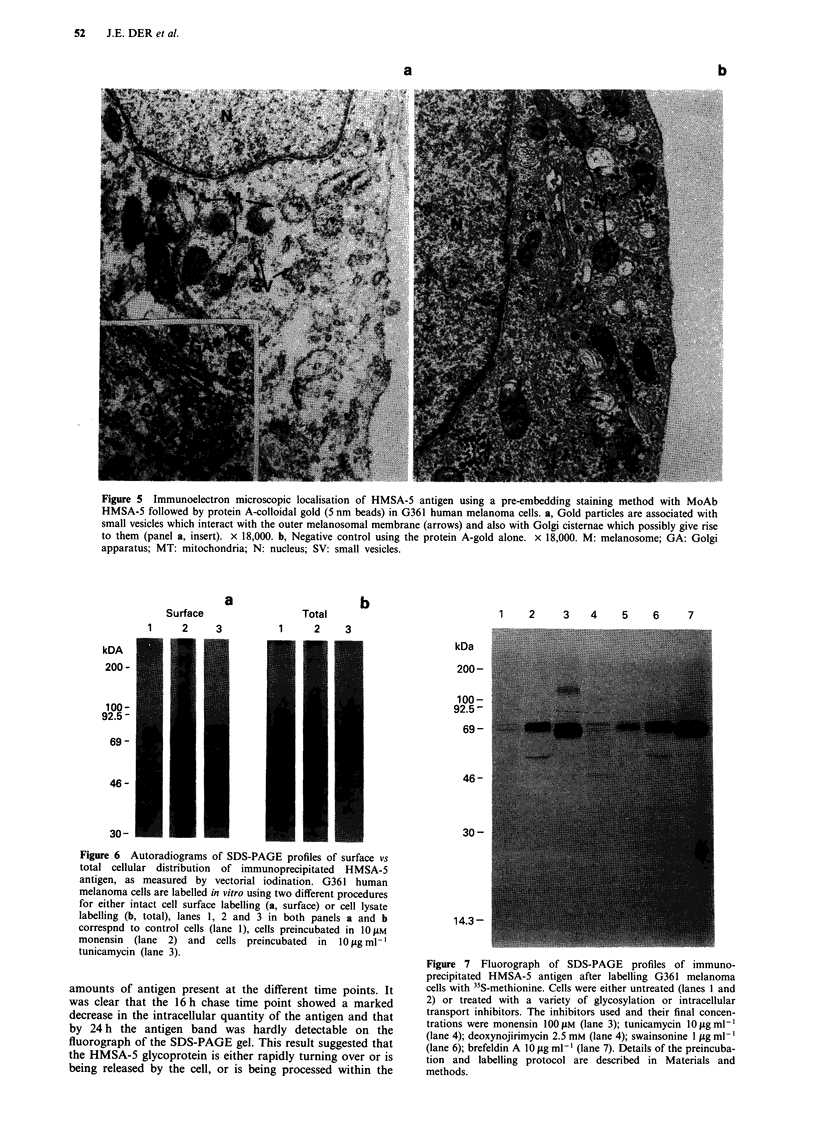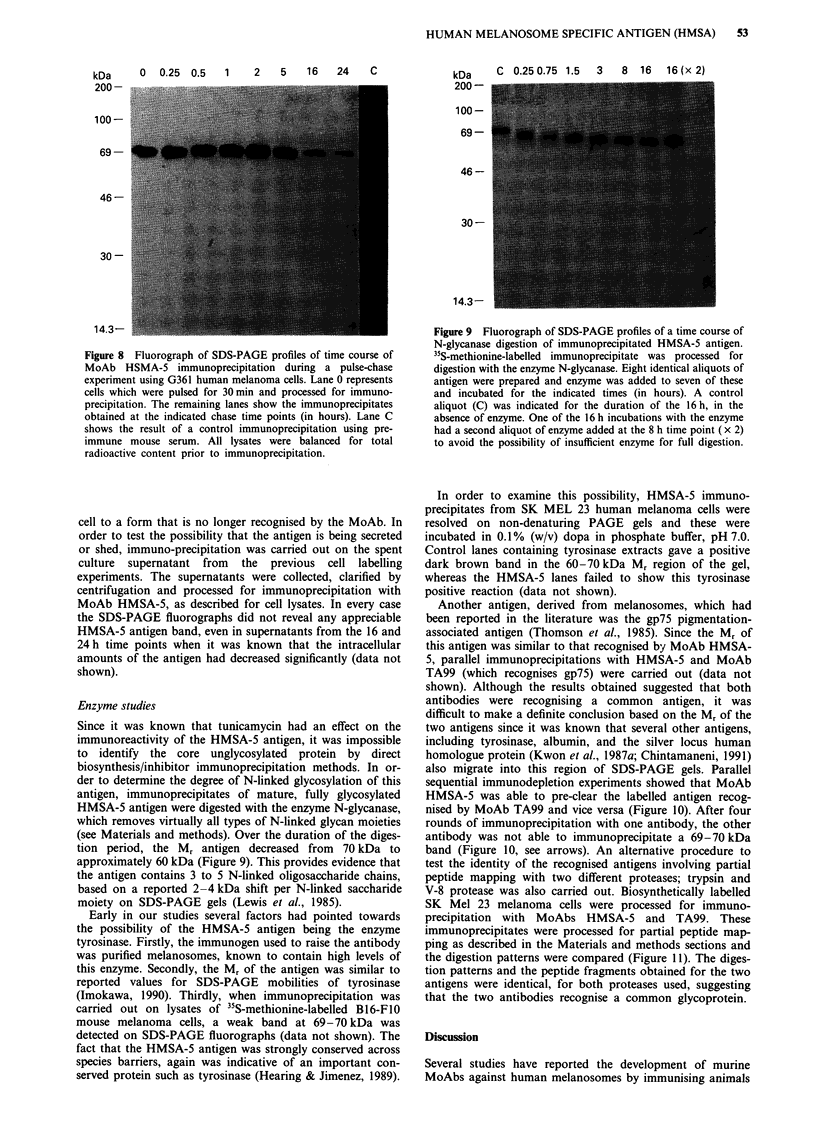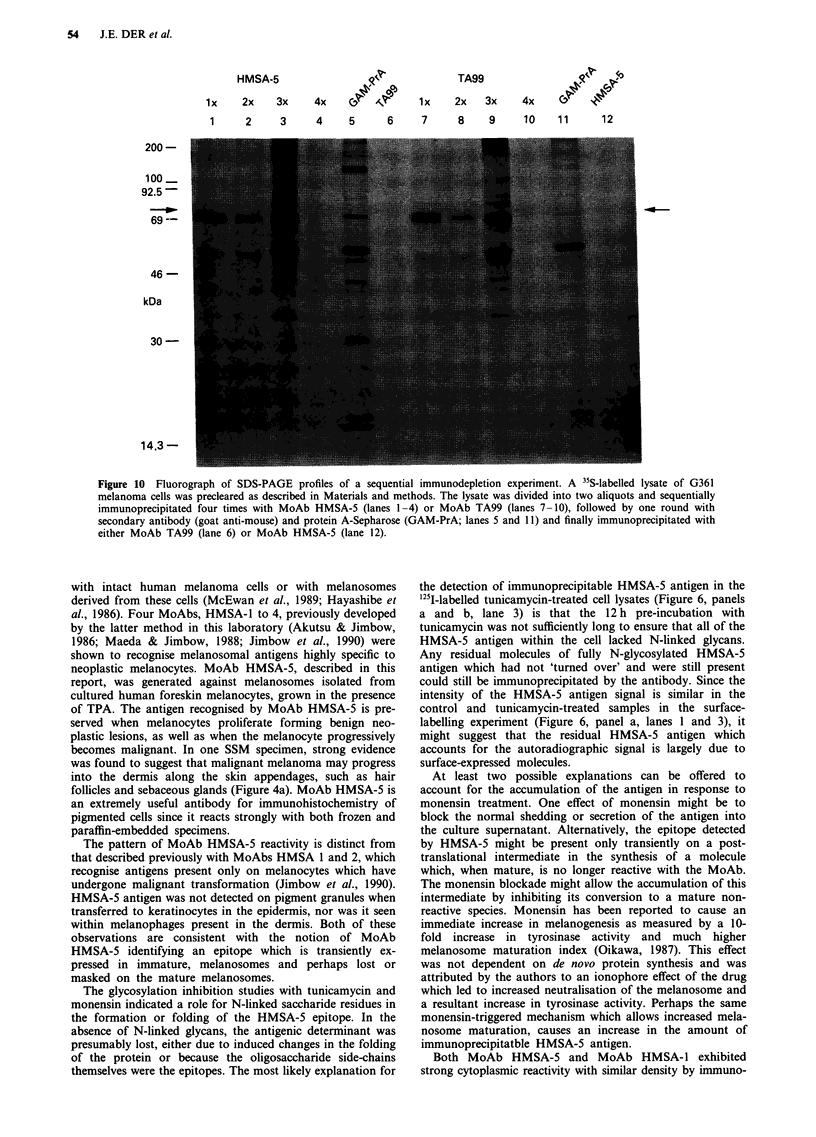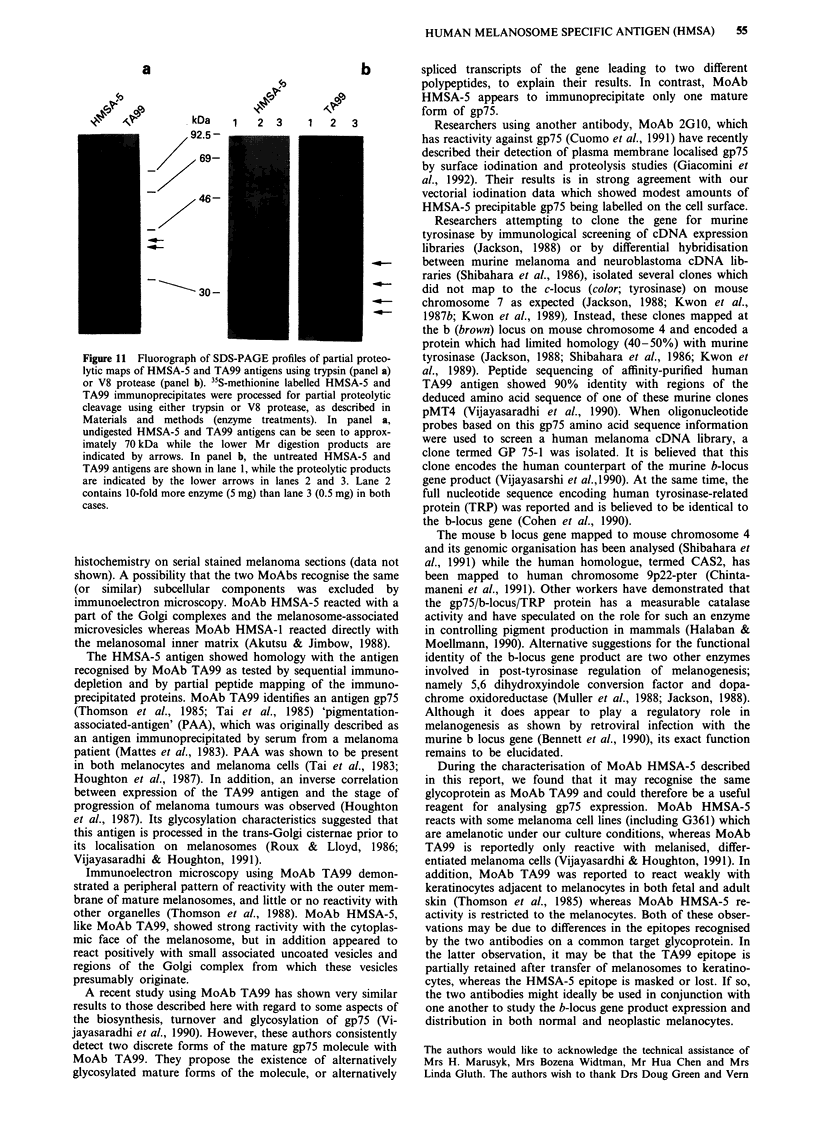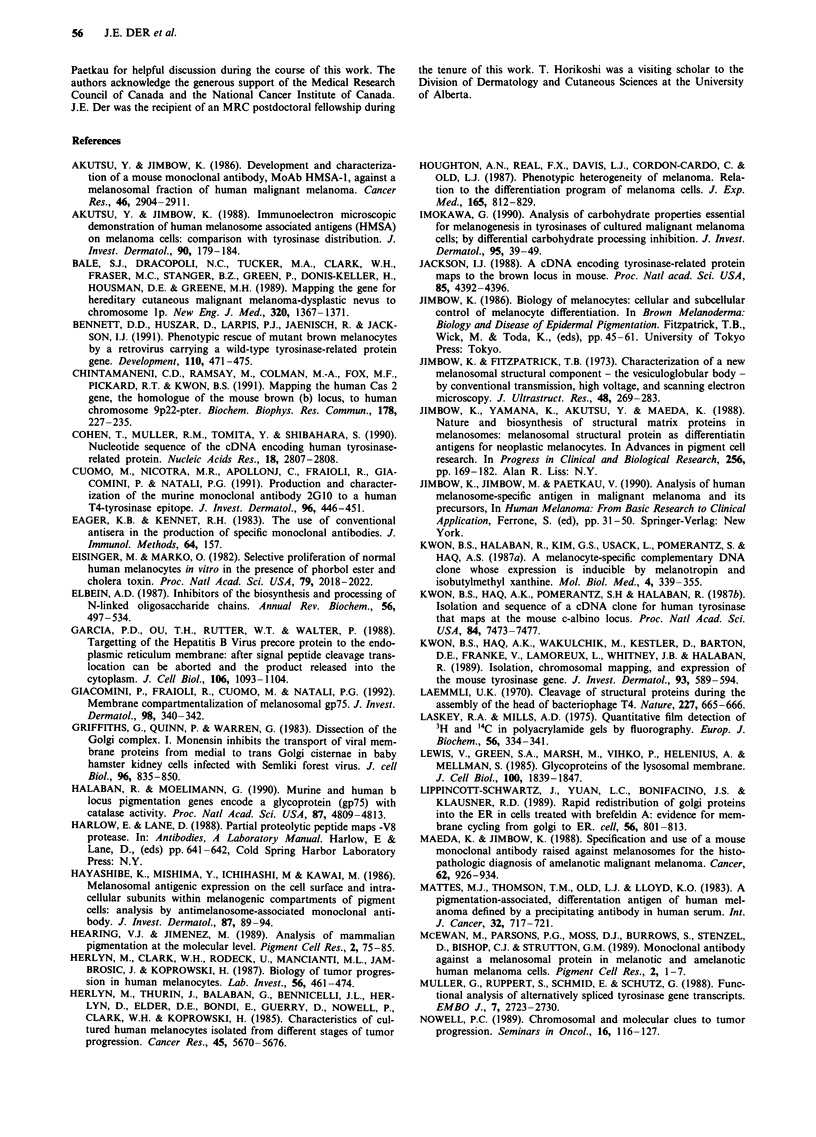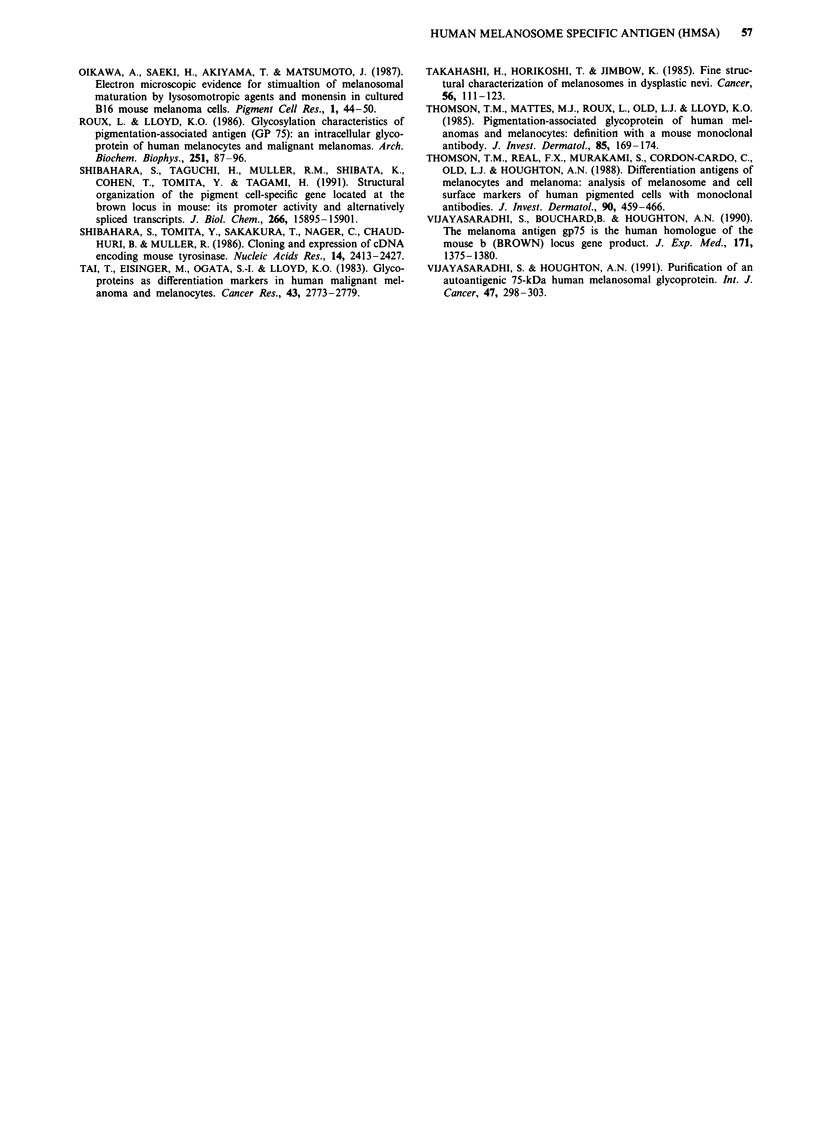# A murine monoclonal antibody, MoAb HMSA-5, against a melanosomal component highly expressed in early stages, and common to normal and neoplastic melanocytes.

**DOI:** 10.1038/bjc.1993.8

**Published:** 1993-01

**Authors:** J. E. Der, W. T. Dixon, K. Jimbow, T. Horikoshi

**Affiliations:** Division of Dermatology and Cutaneous Sciences, University of Alberta, Edmonton, Canada.

## Abstract

**Images:**


Br. J. Cancer (1993), 67, 47–57 © Macmillan Press Ltd., 1993

### A murine monoclonal antibody, MoAb HMSA-5, against a melanosomal component highly expressed in early stages, and common to normal and neoplastic melanocytes

J.E. Der, W.T. Dixon, K. Jimbow & T. Horikoshi

*Division of Dermatology and Cutaneous Sciences, University of Alberta, Edmonton, Alberta, Canada T6G 2S2.*

**Summary** The melanosome is a secretory organelle unique to the melanocyte and its neoplastic counterpart, malignant melanoma. The synthesis and assembly of these intracytoplasmic organelles is not yet fully understood. We have developed a murine monoclonal antibody (MoAb) against melanosomes isolated from human melanocytes (newborn foreskin) cultured in the presence of 12-*O* tetradecanoyl phorbol-13-acetate (TPA). This MoAb, designated HMSA-5 (Human Melanosome-Specific Antigen-5) (IgG1), recognised a cytoplasmic antigen in both normal human melanocytes and neoplastic cells, such as common and dysplastic melanocytic nevi, and malignant melanoma. None of the carcinoma or sarcoma specimens tested showed positive reactivity with MoAb HMSA-5. Under immunoelectron microscopy, immuno-gold deposition was seen on microvesicles associated with melanosomes, and a portion of the ER-Golgi complexes. Radioimmunoprecipitation analysis showed that the HMSA-5 reactive antigen was a glycoprotein of $M_r$ 69 to 73 kDa. A pulse-chase time course study showed that the amount of antigen detected by MoAb HMSA-5 decreased over a 24 h period without significant expression on the cell surface, or corresponding appearance of the antigen in the culture supernatant. This glycoprotein appears to play a role in the early stages of melanosomal development, and the HMSA-5 reactive epitope may be lost during subsequent maturation processes. Importantly, HMSA-5 can be identified in all forms of human melanocytes, hence it can be considered a new common melanocytic marker even on routine paraffin sections.

Human melanocytes migrate from the neural crest into the skin during the first 6 to 8 weeks of embryonic development. During the process of differentiation melanocytes sythesise unique secretory granules, the melanosomes, which are known to contain structural matrix proteins, lipids, melanin, tyrosinase, and some post-tyrosinase regulatory factors (PTRF) (Jimbow, 1986). Mechanisms involved in the synthesis and assembly of these melanosomal constituents have not yet been definitively elucidated. However, an understanding of these processes may be important in determining the molecular events involved in normal melanisation. It may be equally important for an explanation as to why melanosomes found in melanoma cells and their precursors exhibit markedly aberrant morphology (Takahashi *et al.*, 1985). Since a major role of the melanosome is to contain the otherwise toxic and/or tumour-promoting chemical intermediates of melanogenesis (Jimbow, 1986), any process which compromises the structural integrity or correct functioning of the melanosome could have serious consequences to the cell. Previous studies have shown that melanosomes isolated from neoplastic melanocytes express unique antigenic determinants, such as those detected by monoclonal antibodies (MoAbs) HMSA-1 and 2 (Akutsu & Jimbow, 1986; Maeda & Jimbow, 1988). At the molecular level, changes responsible for the malformation may be genetic abnormalities found in transformed cells (Jimbow *et al.*, 1988). Progressive aneuploidy and non-random chromosomal abnormalities involving chromosomes 1, 6 and 7 have been frequently found in melanoma cells (Bale *et al.*, 1989; Herlyn *et al.*, 1987; Herlyn *et al.*, 1985; Nowell, 1989). These changes may result in aberrant expression of gene(s) involved in the biosynthesis of melanosomal constituents, which may lead directly or indirectly to alterations in the structure and/or assembly of melanosomes. Disturbed rates of transcription and putative alternative splicing of mRNAs have also been reported in tumour cells and may contribute to the melanosomal derangement. In addition, signals targeting the transport of proteins to intracellular organelles (Garcia *et al.*, 1988) may be disturbed due to altered co- and/or post-translational modifications. In order to substantiate such putative pathophysiological processes, a comparison of melanosomal constituents found in normal and neoplastic melanocytes is required. We have shown previously that murine MoAbs HMSA-1 and HMSA-2 recognise epitopes on structural matrix proteins highly specific to melanosomes of neoplastic human melanocytes (Akutsu & Jimbow, 1986; Maeda & Jimbow, 1988). In the present study, we have developed a new murine MoAb, designated HMSA-5 ($IgG_1$), against melanosomes isolated from human melanocytes of newborn foreskin, grown in the presence of TPA. We found that MoAb HMSA-5 reacted with normal and neoplastic melanocytes on both paraffin and frozen specimens. Immunoelectron microscopy and immunoprecipitation studies suggested that the HMSA-5 antigen may be a precursor molecule of a melanosomal constituent, associated with microvesicles which give rise to, or fuse with melanosomes, and that the HMSA-5 reactive epitope is lost during the melanosomal differentiation.

Correspondence: K. Jimbow, Division of Dermatology and Cutaneous Sciences, 260G Clinical Wing, Heritage Medical Research Centre, University of Alberta, Edmonton, Alberta, Canada T6G 2S2.
Received 3 April 1992; and in revised form 7 August 1992.

### Materials and methods

### *Cells*

*Human melanocytes* Human melanocytes ($10^8$) were obtained from newborn foreskin and grown under the conditions reported by Eisinger and Marko (1982). Briefly, newborn foreskins were incubated in a trypsin solution, 0.25% (w/v) (Gibso, Grand Island, NY), 12 h at 4°C. The epidermis was then gently peeled from the dermis. The epidermal sheet was vigorously pipetted until a single cell suspension was generated. The suspension was centrifuged and the pellet was resuspended in culture medium supplemented with TPA (Sigma Chemical Co., St. Louis, Mo), 10 ng $ml^{-1}$, and 7.5% (v/v) of foetal calf serum (FCS, Gibco, Grand Island, NY). If fibroblast contamination was found to be present, a single dose of geneticin, 100 $\mu g\ ml^{-1}$ was added to the medium (usually at 3–4 weeks).

*Melanoma cells* Cultured cells of five melanoma cell lines were used in this study. These included G361 (ATCC-1640), HMV II (kindly supplied by Dr T. Kasuga, Tokyo Medical and Dental School, Tokyo, Japan) and SK-Mel 23, SK-Mel 28 and SK-Mel 118 (kindly supplied by Dr A. Houghton, Sloan Kettering Cancer Center, NY).

### Enzymes

N-Glycanase (peptide: N-glycosidase F,E.C. 3.2.2.18) was obtained from Genzyme (Boston, MA). Trypsin (sequencing grade E.C. 3.4.21.4) and V8 protease (endoproteinase glu-C, E.C. 3.4.21.29) were obtained from Boehringer Mannheim (Dorval, Quebec).

### Antibodies

Monoclonal antibody MoAb TA99 (Mel-5) was obtained from Signet Laboratories Inc., Dedham, MA, and has been described previously (Thomson *et al.*, 1985). Normal mouse serum conjugated to cross-linked agarose was obtained from Sigma Chemical Co., St. Louis, Mo. Polyvalent goat anti-mouse antibody was purchased from Cappel, West Chester, PA.

### Chemicals and radiochemicals

Tunicamycin and monensin were obtained from Sigma and were dissolved in DMSO ($10\,mg\,ml^{-1}$) and 95% (v/v) ethanol (10 mM) respectively. Deoxynojirimycin and swainsonine were obtained from Boehringer Mannheim, Dorval, Quebec and were dissolved in a sterile water at $10\,mg\,ml^{-1}$ and $500\,\mu g\,ml^{-1}$ respectively. Brefeldin A was obtained from Bio/Can Scientific, Mississauga, Ontario and was dissolved in 95% (v/v) ethanol at $10\,mg\,ml^{-1}$. $^{125}I$-sodium iodide (506.9 $MBq\,\mu g^{-1}$) was obtained from Amersham (Oakville, Ontario) and L-$^{35}S$-methionine ($29.6\,TBq\,mmol^{-1}$) was from NEN/DUPONT (Mississauga, Ontaria).

### Development of monoclonal antibody

*Immunogen* Approximately $8 \times 10^7$ cultured human melanocytes were collected and stored at $-90°C$. These cells were homogenised in 0.05 M phosphate buffer, pH 6.8, supplemented with 0.25 M sucrose, 20 mM KCl, 4 mM $MgCl_2$, and 1 mM phenylmethyl-sulfonyl fluoride (PMSF). Melanosomes were isolated from an homogenate of these cells by a discontinuous sucrose density gradient ultracentrifugation procedure as described previously (Jimbow *et al.*, 1988). The purity of the melanosomal fraction was examined by both light and electron microscopy.

*Immunisation* The purified human melanosomes were resuspended in phosphate buffered saline (PBS), at an approximate concentration of $1.5\,mg\,ml^{-1}$, sterilised by irradiation with a bacteriostatic dose of 2,500 rad, emulsified with 1.5 ml Freund's complete adjuvant (Gibco, Grand Island, NY), and injected subcutaneously into BALB/c mice (6 to 8 weeks old, female). After three boost injections at 2 week intervals, a test bleed was carried out. The test serum showed a positive reaction with melanocytes cultured in 96 microwell plates at a dilution of 1 in 10,000 with avidin biotin immunoperoxidase complex (ABC) stain (Vectastain ABC kit, Vector Laboratories, Burlingham, CA). A clone, HMSA-5, was selected from a fusion of the immunised splenocytes with murine myeloma cells (Sp2/0), at a 1 to 5 ratio, following a published hybridoma fusion/selection protocol (Eager & Kennet, 1983). For screening of the hybridoma clones, melanocytes cultured in 96 microwell plates (Costar, Cambridge, MA) and paraffin-embedded sections of normal human skin were stained with cultured hybridoma supernatant, using the ABC method.

### Immunostaining

Paraffin embedded sections were deparaffinised in xylene, and rehydrated in a graded series of alcohol solutions. The endogenous peroxidase activity was blocked by incubating sections with 0.3% (v/v) hydrogen peroxide in absolute methanol for 30 min. Sections were washed in PBS and blocked with 1% (w/v) bovine serum albumin (BSA) for 30 min. After a 2 h incubation with hybridoma-culture superntant, sections were washed in PBS and incubated with the biotinylated horse anti-mouse IgG antibody for 1 h. Sections were washed in PBS for 5 min, incubated for 1 h with ABC and the products of the reaction were detected using diamino-benzidine (DAB) as described previously (Akutsu & Jimbow, 1986).

### Radiolabelling

In vivo *biosynthesis labelling* Melanoma cells, G361 or SK Mel-23 ($1 \times 10^6$), in exponential growth phase were cultured in methionine-free media (Select-amine, Gibco, Grand Island, NY) for 2 h, and incubated with medium containing $^{35}S$-methionine (0.5 mCi, Amersham, Arlington Heights, Il), in 5% (v/v) $CO_2$ at 37°C, for 12 h. Cells were harvested by scraping and the cell pellet was lysed with a lysis buffer consisting of 1 mM $MgCl_2$, 0.25% Trasylol (Sigma Chemical Co. St. Louis, Mo), 1% (v/v) NP-40 0.1% (w/v) sodium azide, and 10 mM PMSF in 10 mM Tris-HCl, pH 7.6, for 30 min on ice and processed for immunoprecipitation. When inhibitors of glycosylation/intracellular transport were used for metabolic labelling studies, the cells were pre-incubated with the inhibitors for 6 h and the same concentrations of inhibitors were maintained during the labelling incubation itself. The final concentrations of the inhibitors which were used are described in the figure legends.

In vitro *cell surface labelling* To a suspension (1 ml) of G361 melanoma cells ($5 \times 10^6$ cells $ml^{-1}$) 0.5 mCi of $^{125}I$-sodium iodide was added, and placed on ice. The suspension was then transferred to Iodo-gen coated tubes (Pierce, Terochem, Edmonton, Alberta) and incubated for 5 min on ice. The reaction was terminated by transferring the labelled cell suspension to cold sodium iodide (1 mM) containing media and washed by three successive rounds of centrifugation and resuspension in fresh cold media.

*Labelling of cell lysates* Alternatively, the cell pellet of G361 melanoma cells ($5 \times 10^6$) was incubated with 1 ml lysis buffer (1 ml) for 30 min at 4°C. The lysate was centrifuged (16,000 *g*, 30 min) and transferred to a fresh tube, to which $^{125}I$-sodium iodide (0.5 mCi) was added The labelling mixture was then transferred to an Iodo-gen coated tube, incubated for 5 min on ice, and applied to a PD-10 column (Pharmacia, Baie D'Urfé, Québec), pre-equilibrated with a wash buffer (0.5 M NaCl, 5 mM EDTA 1% [w/v] NP 40 and 1% [v/v] FCS in 50 mM Tris HCl, pH 7.5). Radiolabelled protein was eluted from the column with the same buffer (without FCS) and fractions were collected (1.0 ml/fraction) and aliquots counted by liquid scintillation counting. The radioactive protein peak fractions were pooled and processed for immunoprecipitation.

### Immunoprecipitation

The supernatants of cell lysates (from *in vivo* biosynthetic labelling of cell surface iodination or from whole cell lysate iodination) were diluted to 10 ml with heat-inactivated FCS solution supplemented with 0.25% (v/v) Trasylol, 0.1% (w/v) sodium azide, 10 mM PMSF and 1% (v/v) NP-40. The diluted mixtures were precleared with pre-immune mouse serum agarose beads (Sigma Chemical Co., St. Louis, Mo), 50 µl of 50% (w/v) suspension three times and then with protein-A Sepharose (Pharmacia, Baie D'Urfe, Que), 50 µl of 50% (w/v) suspension. The precleared lysates were then incubated with cultured supernatant (50 µl) or purified antibody (10 µl) by end-over-end rotation at 4°C for 10 h, followed by incubation with a polyvalent goat anti-mouse antibody (Cappel, West Chester, PA), 10 µl for 4 h, and finally with protein-A Sepharose beads, 50 µl for 2 h. The beads containing the immune complex were washed with wash buffer I (0.5 M NaCl, 5 mM EDTA, 1% [v/v] NP-40, in 50 mM Tris-HCl, pH 7.5), 10 ml, four times, and with wash buffer II (0.5 M NaCl and 5 mM EDTA in 50 mM Tris-HCl, pH 7.5) 10 ml, twice. The samples were boiled for 5 min in SDS-PAGE sample buffer and resolved on 15% (w/v) polyacrylamide gels (SDS-PAGE) (Laemmli, 1970). For semi-quantitative studies, cell lysates were balanced for radioactive content. Briefly, aliquots of the cell lysates (20 µl) were precipitated with 25% (w/v) TCA (5 ml), on ice for 30 min, absorbed on filter paper and processed for liquid scintillation counting. Aliquots of cell lysates with equal radioactivity were processed for immunoprecipitation.

*Biosynthetic labelling studies*

*Pulse chase time course* Following a 2 h methionine depletion, G361 melanoma cells were pulse-labelled for 30 min with media containing $^{35}$S-methionine. The radioactive media was then removed and replaced with unlabelled culture medium and the cells were incubated for various times. Cells were harvested at 0, 0.25, 0.5, 1, 2, 5, 16 and 24 h time intervals during the chase period, and processed for immunoprecipitation with MoAb HMSA-5 as described above, and resolved on 15% SDS-PAGE gels (Laemmli, 1970).

*Sequential immunodepletion study*

An $^{35}$S-labelled lysate of G361 melanoma cells was precleared four times with normal mouse serum agarose and once with protein A-Sepharose as described above, and divided into two identical aliquots. One aliquot was subjected to four sequential rounds of immuno-precipitation with HMSA-5 culture supernatant (50 µl per round) as described above, followed by one round with the secondary antibody alone to absorb any residual HMSA-5 immune complexes. The supernatant from this last step was then immunoprecipitated with MoAb TA99 (10 µl purified MEL-5, Signet Labs.). The other aliquot was processed similarly, except MoAb TA99 was used for the initial four rounds of immunoprecipitation followed by a final round with MoAb HMSA-5. The resulting immunoprecipitates were analysed on SDS-PAGE gels as described below.

*Glycosylation studies*

*N-glycanase digestion* The HMSA-5 immunoprecipitate was washed end-over-end with wash buffer I and II at 4°C, and then boiled in 0.5% SDS for 3 min. Aliquots of the SDS-glycoprotein solution (20 µl) were diluted and incubated with N-Glycanase (Genzyme Corporation, Boston, MA) according to the manufacturer's recommendations for 0.25, 0.75, 1.5, 3, 8 and 16 h at 37°C. The reaction was stopped by the addition of SDS-PAGE sample buffer and the samples were boiled for 5 min and processed for SDS-PAGE analysis. A control digestion was carried out under identical conditions for the longest time period without the addition of enzyme.

*Trypsin digestion*

$^{35}$S-labelled lysates of cultured melanoma cells ($2 \times 10^6$) were immunoprecipitated using culture supernatant of MoAb HMSA-5 (20 µl) or purified MoAb TA99 (Signet Lab Inc. Dedham, MA; 10 µl of a 1 in 30 dilution) as described above. The resulting immunoprecipitates were washed with wash buffers I and II and boiled in 1% SDS, for 5 min. SDS glycoprotein supernatant (50 µl) was mixed with 50 µl of 0.1 M Tris-HCl, pH 8.5, and then treated with 5 µl of trypsin suspended in 10 µl of 1 mM HCl. The reaction was stopped by the addition of SDS-PAGE sample buffer and the samples were prepared for SDS-PAGE analysis.

*V8 protease digestion*

Immunoprecipitates of HMSA-5 and TA99 were resuspended in V8 protease sample buffer containing 0.1% (w/v) SDS, 1 mM EDTA, 2.5 mM DTT in 125 mM Tris-HCl, pH 6.8 and boiled for 5 min. Glycerol was added to a final concentration of 20% (w/v) and the sample supernatants were transferred to the wells of an SDS-PAGE gel to which 1 mM EDTA had been added and which had a stacking gel at least 3 cm in depth. The sample was overlayered with 10 µl of 10% glycerol, 0.1% (w/v) SDS, 1 mM EDTA, 2.5 mM DTT and 0.001% bromophenol blue (BPB) in 125 mM Tris-HCl, pH 6.8, containing 0.05, 0.5 and 5 µg of V8 protease. Electrophoresis was continued until the BPB marker had migrated 2 cm into the stacking gel, after which the power was turned off and the protein was allowed to digest for 30 min. The power was restored and the electrophoresis was continued until the BPB marker had reached the end of the separating gel (Harlow & Lane, 1988). The gel was processed for fluorography to analyse the resulting peptide fragments.

*Gel electrophoresis*

Immunoprecipitates were resuspended in Laemmli sample buffer (two volumes) (Laemmli, 1970). The samples were boiled for 5 min, cooled, and loaded on SDS-PAGE gels containing a 5% (w/v) acrylamide stacking gel and a 15% (w/v) acrylamide separating gel. $^{14}$C-Labelled molecular weight standards (Amersham, Arlington Heights, IL) were used to calibrate the size of proteins. The markers included: myosin (200 kDa), phosphorylase b (100 and 92.5 kDa), bovine serum albumin (69 kDa), ovalbumin (46 kDa), carbonic anhydrase (30 kDa) and lysozyme (14.3 kDa). Gels of $^{125}$I-samples were fixed with water:methanol:acetic acid (68:25:7 by volume) and processed for autoradiography whereas $^{35}$S-methionine containing gels were processed for fluorography (Laskey & Mills, 1975).

*Immunoelectron microscopy*

Subconfluent, monolayer cultures of G361 melanoma cells were washed with PBS, fixed in 4% paraformaldehyde in 0.1 M PBS, pH 7.2, for 45 min. After PBS wash the cells were incubated with 0.2% saponin, for 30 min and then blocked with 1% BSA, and incubated with the primary antibody for 2 h. Cells were washed with PBS and incubated with protein A-gold (5 nm in particle size) (Janssen Biotech N.V., Olen, Belgium) (1 in 50 dilution) for 60 min. They were postfixed with 2.5% glutaraldehyde for 30 min, and osmium tetroxide for 60 min, dehydrated and then embedded in LX-112 resin (Ladd Research Industries, Inc. Burlington, Vermont). Ultrathin sectioning was carried out with a MT-2 Porter Blum ultratome. Sections were examined under a Siemens Elminskop 101.

### Results

*Immunohistochemical study*

*Normal tissues* MoAb HMSA-5 reacted strongly with epidermal melanocytes in both newborn and adult skin of frozen and paraffin-embedded tissues (Figure 1). At a higher-power view, positive-granular immunoreactions were seen in the perikaryon and dendrites of melanocytes. Follicular melanocytes in the bulb also showed a positive reaction with the MoAb (data not shown). In both frozen and paraffin sections, no other cell types besides melanocytes in the skin reacted positively with MoAb HMSA-5. Keratinocytes and melanophages that contained melanosomes were negative for immunoreaction, revealing dark green granules counterstained with methyl green. None of the other normal tissues tested reacted with MoAb HMSA-5 (Table I).

*Benign melanocytic nevi* MoAb HMSA-5 reacted positively with cells of both epidermal and dermal nevi. They showed

**Figure 1** Normal human adult skin stained with MoAb HMSA-5 by the ABC technique, showing melanocytes along the dermal-epidermal junction. The dendritic processes reveal immunoreactive granular materials (× 160).

**Figure 2** Dysplastic melanocytic nevus stained with MoAb HMSA-5, revealing highly positive large epithelioid cells near the dermal-epidermal junction. ABC staining (× 160).

granular cytoplasmic staining similar to that seen in normal epidermal and follicular melanocytes. Dysplastic melanocytic nevi (DMN) reacted strongly with MoAb HMSA-5. In dermal nevi, nests of cells close to the epidermis (which contained numerous melanosomes) reacted more strongly than did those nevus cells that were located in the middle or lower part of the dermis (Figure 2).

*Malignant melanoma* All subtypes of human malignant melanoma tested, e.g. superficial spreading melanoma (SSM), lentigo maligna melanoma (LMM), and nodular melanoma (NM), reacted positively with MoAb HMSA-5. In most melanoma cells, the entire cytoplasm was filled densely with strongly positive granules. In SSM, many large 'pagetoid' cells located within the epidermis reacted with MoAb HMSA-5 (Figure 3). In one SSM specimen a continuous layer of MoAb HMSA-5 positive cells was seen along the basement membrane of hair follicles, sebaceous glands and their ducts (Figure 4).

*Non-melanocytic tumours* None of the tumours tested reacted with MoAb HMSA-5, including neural-crest derived tumours.

*Cultured cells* The reactivity of MoAb HMSA-5 was tested with five human melanoma cell lines which included SK Mel-23, SK Mel-28, SK Mel-118, HMV II, and G361. SK Mel-28 HMV II, and G361 cultured cell lines did not produce much visible melanin pigments under our culture conditions. MoAb HMSA-5 reacted homogenously with HMV II and G361 cell lines. The highly pigmented, SK Mel-23 cells reacted strongly, whereas SK Mel-118 amelanotic melanoma cells showed a much lower reaction with MoAb HMSA-5. In all of the positive-cell lines the immunostaining was granular and located within the cytoplasm.

*Immunoelectron microscopy*

G361 human melanoma cells contained a large number of melanosomes in early stages of development. These consisted of both spherical melanosomes with microvesicular inclusions, and ellipsoidal melanosomes with lamellar internal structure. In certain melanosomes both microvesicular and lamellar matrix were seen. Other organelles within melanoma cells included well developed Golgi complexes, endoplasmic reticulum (ER), and mitochondria. Positive immunogold reactivity were seen on small vesicles (about 20–40 nm) which clustered mostly around early-stage melanosomes. These vesicles appeared to be similar in structure to those

**Table I** Distribution of MoAb HMSA-5 reactive antigen in non-melanocytic and melanocytic tissues and tumours in paraffin sections

| *Tissue* | *Reactivity* | *Tissue* | *Reactivity* |
|---|---|---|---|
| *Normal tissue* | | | |
| Appendix | – (0/2)[a] | Gall bladder | – (0/3) |
| Kidney | – (0/2) | Spleen | – (0/3) |
| Placenta | – (0/1) | Fallopian tube | – (0/2) |
| Prostate | – (0/1) | Lung | – (0/1) |
| Stomach | – (0/3) | Fat tissue | – (0/3) |
| Brain | – (0/3) | Conjunctiva | – (0/1) |
| | | Liver | – (0/2) |
| *Neoplastic Tissue* | | | |
| Astrocytoma | – (0/2) | LMM[b] | + (15/16) |
| Meningioma | – (0/2) | SSM[c] | + (27/29) |
| Morton's neurinoma | – (0/2) | Nodular melanoma | + (2/2) |
| Neurofibroma | – (0/2) | Metastatic melanoma | + (3/4) |
| Neurilemmoma | – (0/2) | Nevi: | |
| Lung carcinoma | – (0/3) | compound type | + (10/10) |
| Colon carcinoma | – (0/1) | junctional type | + (5/5) |
| SCC[d] | – (0/1) | dermal type | + (5/5) |
| Teratoma | – (0/6) | dysplastic type | + (15/15) |

[a]Numbers in parentheses refer to the number of positive specimens/total number of specimens studied. [b]LMM – Lentigo maligna melanoma. [c]SSM – Superficial spreading melanoma. [d]SCC – Squamous cell carcinoma of skin.

**Figure 3** Superficial spreading melanoma (SSM) specimen stained with MoAb HMSA-5, showing nests of positively stained cells aligned along the dermal-epidermal junction. 'Pagetoid' cells in the epidermis also reacted with HMSA-5. (× 160).

**Figure 4** SSM specimen stained with MoAb HMSA-5, revealing melanocytes located in the hair follicles (HF) and sebaceous glands (SG) as a continuous layer **a**. Higher magnification of a serial section through the same SSM specimen stained with HMSA-5, showing positively stained tumour cell nests (TN) adjacent to the continuous layer of melanocytes (MC) bordering the sebaceous gland and hair follicles. Melanophages (MP) are stained dark, but they are negative **b**.

previously reported as vesiculoglobular bodies or microvesicles within the melanosomes (Jimbow & Fitzpatrick, 1973). Immunogold particles were also seen on part of the Golgi-ER complex while the nucleus, mitochondria, rough ER, and most of the coated vesicles did not show a positive immunogold reaction (Figure 5).

### *Antigen characterisation and the use of inhibitors of protein glycosylation*

MoAb HMSA-5 immunoprecipitates from $^{125}$I-labelled lysates of either surface-labelled or total cell homogenates of G361 cells revealed a band with an approximate $M_r$ of 70 kDa on SDS-PAGE gels (Figure 6, panels a and b, lane 1). Relatively small amounts of antigen were detected after the surface labelling (Figure 6, panel a, lane 1). This was consistent with flow cytometry measurements of cell surface HMSA-5 antigen expression using a fluorescein-labelled secondary antibody (data not shown). When different melanoma cell lines were compared, some small variations in the $M_r$ of the antigen were seen on SDS-PAGE gels, presumably due to different degrees of post-translational modification.

When immunoprecipitation experiments were carried out on cell lysates of G361 cells pre-treated for 12 h with the glycosylation inhibitors monensin or tunicamycin, some differences in the autoradiographic gel profiles were seen compared to controls, despite the fact that all experiments were balanced for total radioactivity (see Materials and methods). Monensin is a carboxylic acid ionophore which is known to partially inhibit post-translational modifications within the Golgi complex by disrupting proton gradients necessary for vesicular transport (Griffiths *et al.*, 1983). Monensin caused an apparent increase in the amount of immunoprecipitable antigen compared to controls in both surface and total labelled cell lysates (Figure 6, panels a and b, lane 2). Coincident with this apparent quantitative increase was a slight decrease in the $M_r$ of the immunoprecipitated antigen, consistent with alterations in post-translational processing. Tunicamycin is an analogue of N-acetyl-glucosamine which prevents the correct assembly of oligosaccharide-lipids necessary for co-translational N-glycosylation (Elbein, 1987). When this inhibitor was used, there was a decrease in the amount of immunoprecipitable antigen, particularly in the total-labelled lysate immunoprecipitate (Figure 6, panel b, lane 3) without an accompanying downward shift in the $M_r$ on SDS-PAGE.

Similar inhibitor experiments were carried out using a biosynthetic radiolabelling protocol in which the inhibitors were present during a 6 h pre-incubation as well as during a 6 h labelling period with $^{35}$S-methionine. When these experiments were carried out and immunoprecipitates from lysates balanced for total radioactivity were compared, monensin again caused a slight decrease in the $M_r$ of the HMSA-5 antigen and a quantitative increase in its amount compared to control (Figure 7, lanes 2 and 3). However, when tunicamycin was used, MoAb HMSA-5 was unable to immunoprecipitate any proteins detectable by SDS-PAGE fluorography (Figure 7, lane 4).

Neither deoxynojirimycin or swainsonine, inhibitors of early and late N-linked glycan processing (glucosidase 1 and α-mannosidase II, respectively) (Elbein, 1987) had significant effects on the $M_r$ of the HMSA-5 antigen, although the amount of immunoprecipitable antigen was decreased compared to untreated cells (Figure 7, lanes 5 and 6). Brefeldin A, which perturbs intracellular transport by blocking ER to Golgi transport (Lippincott-Schwartz *et al.*, 1989) gave a gel profile for the antigen which is very similar to that seen with monensin, i.e., a net accumulation of a slightly smaller $M_r$ form of the molecule (Figure 7, lane 7).

### *Pulse-chase experiments*

A pulse-chase time course experiment using $^{35}$S-methionine was carried out to follow the biosynthesis of the HMSA-5 antigen. The antigen was readily precipitable at the 30 min pulse (0 h chase) time point and had a $M_r$ of 68–69 kDa (Figure 8). There was a very slight increase (1–2kDa) in the $M_r$ of the antigen over the first hour of the chase, after which time the molecule maintained its $M_r$ for the duration of the time course. Since the cell lysates were balanced for radioactivity, it was possible to compare semi-quantitatively the

**Figure 5** Immunoelectron microscopic localisation of HMSA-5 antigen using a pre-embedding staining method with MoAb HMSA-5 followed by protein A-colloidal gold (5 nm beads) in G361 human melanoma cells. **a**, Gold particles are associated with small vesicles which interact with the outer melanosomal membrane (arrows) and also with Golgi cisternae which possibly give rise to them (panel **a**, insert). × 18,000. **b**, Negative control using the protein A-gold alone. × 18,000. M: melanosome; GA: Golgi apparatus; MT: mitochondria; N: nucleus; SV: small vesicles.

**Figure 6** Autoradiograms of SDS-PAGE profiles of surface *vs* total cellular distribution of immunoprecipitated HMSA-5 antigen, as measured by vectorial iodination. G361 human melanoma cells are labelled *in vitro* using two different procedures for either intact cell surface labelling (**a**, surface) or cell lysate labelling (**b**, total), lanes 1, 2 and 3 in both panels **a** and **b** correspnd to control cells (lane 1), cells preincubated in 10 μM monensin (lane 2) and cells preincubated in 10 μg $ml^{-1}$ tunicamycin (lane 3).

amounts of antigen present at the different time points. It was clear that the 16 h chase time point showed a marked decrease in the intracellular quantity of the antigen and that by 24 h the antigen band was hardly detectable on the fluorograph of the SDS-PAGE gel. This result suggested that the HMSA-5 glycoprotein is either rapidly turning over or is being released by the cell, or is being processed within the

**Figure 7** Fluorograph of SDS-PAGE profiles of immunoprecipitated HMSA-5 antigen after labelling G361 melanoma cells with $^{35}S$-methionine. Cells were either untreated (lanes 1 and 2) or treated with a variety of glycosylation or intracellular transport inhibitors. The inhibitors used and their final concentrations were monensin 100 μM (lane 3); tunicamycin 10 μg $ml^{-1}$ (lane 4); deoxynojirimycin 2.5 mM (lane 4); swainsonine 1 μg $ml^{-1}$ (lane 6); brefeldin A 10 μg $ml^{-1}$ (lane 7). Details of the preincubation and labelling protocol are described in Materials and methods.

**Figure 8** Fluorograph of SDS-PAGE profiles of time course of MoAb HSMA-5 immunoprecipitation during a pulse-chase experiment using G361 human melanoma cells. Lane 0 represents cells which were pulsed for 30 min and processed for immunoprecipitation. The remaining lanes show the immunoprecipitates obtained at the indicated chase time points (in hours). Lane C shows the result of a control immunoprecipitation using pre-immune mouse serum. All lysates were balanced for total radioactive content prior to immunoprecipitation.

**Figure 9** Fluorograph of SDS-PAGE profiles of a time course of N-glycanase digestion of immunoprecipitated HMSA-5 antigen. $^{35}$S-methionine-labelled immunoprecipitate was processed for digestion with the enzyme N-glycanase. Eight identical aliquots of antigen were prepared and enzyme was added to seven of these and incubated for the indicated times (in hours). A control aliquot (C) was indicated for the duration of the 16 h, in the absence of enzyme. One of the 16 h incubations with the enzyme had a second aliquot of enzyme added at the 8 h time point (× 2) to avoid the possibility of insufficient enzyme for full digestion.

cell to a form that is no longer recognised by the MoAb. In order to test the possibility that the antigen is being secreted or shed, immuno-precipitation was carried out on the spent culture supernatant from the previous cell labelling experiments. The supernatants were collected, clarified by centrifugation and processed for immunoprecipitation with MoAb HMSA-5, as described for cell lysates. In every case the SDS-PAGE fluorographs did not reveal any appreciable HMSA-5 antigen band, even in supernatants from the 16 and 24 h time points when it was known that the intracellular amounts of the antigen had decreased significantly (data not shown).

*Enzyme studies*

Since it was known that tunicamycin had an effect on the immunoreactivity of the HMSA-5 antigen, it was impossible to identify the core unglycosylated protein by direct biosynthesis/inhibitor immunoprecipitation methods. In order to determine the degree of N-linked glycosylation of this antigen, immunoprecipitates of mature, fully glycosylated HMSA-5 antigen were digested with the enzyme N-glycanase, which removes virtually all types of N-linked glycan moieties (see Materials and methods). Over the duration of the digestion period, the $M_r$ antigen decreased from 70 kDa to approximately 60 kDa (Figure 9). This provides evidence that the antigen contains 3 to 5 N-linked oligosaccharide chains, based on a reported 2–4 kDa shift per N-linked saccharide moiety on SDS-PAGE gels (Lewis *et al.*, 1985).

Early in our studies several factors had pointed towards the possibility of the HMSA-5 antigen being the enzyme tyrosinase. Firstly, the immunogen used to raise the antibody was purified melanosomes, known to contain high levels of this enzyme. Secondly, the $M_r$ of the antigen was similar to reported values for SDS-PAGE mobilities of tyrosinase (Imokawa, 1990). Thirdly, when immunoprecipitation was carried out on lysates of $^{35}$S-methionine-labelled B16-F10 mouse melanoma cells, a weak band at 69–70 kDa was detected on SDS-PAGE fluorographs (data not shown). The fact that the HMSA-5 antigen was strongly conserved across species barriers, again was indicative of an important conserved protein such as tyrosinase (Hearing & Jimenez, 1989).

In order to examine this possibility, HMSA-5 immunoprecipitates from SK MEL 23 human melanoma cells were resolved on non-denaturing PAGE gels and these were incubated in 0.1% (w/v) dopa in phosphate buffer, pH 7.0. Control lanes containing tyrosinase extracts gave a positive dark brown band in the 60–70 kDa $M_r$ region of the gel, whereas the HMSA-5 lanes failed to show this tyrosinase positive reaction (data not shown).

Another antigen, derived from melanosomes, which had been reported in the literature was the gp75 pigmentation-associated antigen (Thomson *et al.*, 1985). Since the $M_r$ of this antigen was similar to that recognised by MoAb HMSA-5, parallel immunoprecipitations with HMSA-5 and MoAb TA99 (which recognises gp75) were carried out (data not shown). Although the results obtained suggested that both antibodies were recognising a common antigen, it was difficult to make a definite conclusion based on the $M_r$ of the two antigens since it was known that several other antigens, including tyrosinase, albumin, and the silver locus human homologue protein (Kwon *et al.*, 1987*a*; Chintamaneni, 1991) also migrate into this region of SDS-PAGE gels. Parallel sequential immunodepletion experiments showed that MoAb HMSA-5 was able to pre-clear the labelled antigen recognised by MoAb TA99 and vice versa (Figure 10). After four rounds of immunoprecipitation with one antibody, the other antibody was not able to immunoprecipitate a 69–70 kDa band (Figure 10, see arrows). An alternative procedure to test the identity of the recognised antigens involving partial peptide mapping with two different proteases; trypsin and V-8 protease was also carried out. Biosynthetically labelled SK Mel 23 melanoma cells were processed for immunoprecipitation with MoAbs HMSA-5 and TA99. These immunoprecipitates were processed for partial peptide mapping as described in the Materials and methods sections and the digestion patterns were compared (Figure 11). The digestion patterns and the peptide fragments obtained for the two antigens were identical, for both proteases used, suggesting that the two antibodies recognise a common glycoprotein.

### Discussion

Several studies have reported the development of murine MoAbs against human melanosomes by immunising animals

**Figure 10** Fluorograph of SDS-PAGE profiles of a sequential immunodepletion experiment. A $^{35}$S-labelled lysate of G361 melanoma cells was precleared as described in Materials and methods. The lysate was divided into two aliquots and sequentially immunoprecipitated four times with MoAb HMSA-5 (lanes 1–4) or MoAb TA99 (lanes 7–10), followed by one round with secondary antibody (goat anti-mouse) and protein A-Sepharose (GAM-PrA; lanes 5 and 11) and finally immunoprecipitated with either MoAb TA99 (lane 6) or MoAb HMSA-5 (lane 12).

with intact human melanoma cells or with melanosomes derived from these cells (McEwan *et al.*, 1989; Hayashibe *et al.*, 1986). Four MoAbs, HMSA-1 to 4, previously developed by the latter method in this laboratory (Akutsu & Jimbow, 1986; Maeda & Jimbow, 1988; Jimbow *et al.*, 1990) were shown to recognise melanosomal antigens highly specific to neoplastic melanocytes. MoAb HMSA-5, described in this report, was generated against melanosomes isolated from cultured human foreskin melanocytes, grown in the presence of TPA. The antigen recognised by MoAb HMSA-5 is preserved when melanocytes proliferate forming benign neoplastic lesions, as well as when the melanocyte progressively becomes malignant. In one SSM specimen, strong evidence was found to suggest that malignant melanoma may progress into the dermis along the skin appendages, such as hair follicles and sebaceous glands (Figure 4a). MoAb HMSA-5 is an extremely useful antibody for immunohistochemistry of pigmented cells since it reacts strongly with both frozen and paraffin-embedded specimens.

The pattern of MoAb HMSA-5 reactivity is distinct from that described previously with MoAbs HMSA 1 and 2, which recognise antigens present only on melanocytes which have undergone malignant transformation (Jimbow *et al.*, 1990). HMSA-5 antigen was not detected on pigment granules when transferred to keratinocytes in the epidermis, nor was it seen within melanophages present in the dermis. Both of these observations are consistent with the notion of MoAb HMSA-5 identifying an epitope which is transiently expressed in immature, melanosomes and perhaps lost or masked on the mature melanosomes.

The glycosylation inhibition studies with tunicamycin and monensin indicated a role for N-linked saccharide residues in the formation or folding of the HMSA-5 epitope. In the absence of N-linked glycans, the antigenic determinant was presumably lost, either due to induced changes in the folding of the protein or because the oligosaccharide side-chains themselves were the epitopes. The most likely explanation for the detection of immunoprecipitable HMSA-5 antigen in the $^{125}$I-labelled tunicamycin-treated cell lysates (Figure 6, panels a and b, lane 3) is that the 12 h pre-incubation with tunicamycin was not sufficiently long to ensure that all of the HMSA-5 antigen within the cell lacked N-linked glycans. Any residual molecules of fully N-glycosylated HMSA-5 antigen which had not 'turned over' and were still present could still be immunoprecipitated by the antibody. Since the intensity of the HMSA-5 antigen signal is similar in the control and tunicamycin-treated samples in the surface-labelling experiment (Figure 6, panel a, lanes 1 and 3), it might suggest that the residual HMSA-5 antigen which accounts for the autoradiographic signal is largely due to surface-expressed molecules.

At least two possible explanations can be offered to account for the accumulation of the antigen in response to monensin treatment. One effect of monensin might be to block the normal shedding or secretion of the antigen into the culture supernatant. Alternatively, the epitope detected by HMSA-5 might be present only transiently on a post-translational intermediate in the synthesis of a molecule which, when mature, is no longer reactive with the MoAb. The monensin blockade might allow the accumulation of this intermediate by inhibiting its conversion to a mature non-reactive species. Monensin has been reported to cause an immediate increase in melanogenesis as measured by a 10-fold increase in tyrosinase activity and much higher melanosome maturation index (Oikawa, 1987). This effect was not dependent on *de novo* protein synthesis and was attributed by the authors to an ionophore effect of the drug which led to increased neutralisation of the melanosome and a resultant increase in tyrosinase activity. Perhaps the same monensin-triggered mechanism which allows increased melanosome maturation, causes an increase in the amount of immunoprecipitatble HMSA-5 antigen.

Both MoAb HMSA-5 and MoAb HMSA-1 exhibited strong cytoplasmic reactivity with similar density by immuno-

**Figure 11** Fluorograph of SDS-PAGE profiles of partial proteolytic maps of HMSA-5 and TA99 antigens using trypsin (panel **a**) or V8 protease (panel **b**). $^{35}$S-methionine labelled HMSA-5 and TA99 immunoprecipitates were processed for partial proteolytic cleavage using either trypsin or V8 protease, as described in Materials and methods (enzyme treatments). In panel **a**, undigested HMSA-5 and TA99 antigens can be seen to approximately 70 kDa while the lower Mr digestion products are indicated by arrows. In panel **b**, the untreated HMSA-5 and TA99 antigens are shown in lane 1, while the proteolytic products are indicated by the lower arrows in lanes 2 and 3. Lane 2 contains 10-fold more enzyme (5 mg) than lane 3 (0.5 mg) in both cases.

histochemistry on serial stained melanoma sections (data not shown). A possibility that the two MoAbs recognise the same (or similar) subcellular components was excluded by immunoelectron microscopy. MoAb HMSA-5 reacted with a part of the Golgi complexes and the melanosome-associated microvesicles whereas MoAb HMSA-1 reacted directly with the melanosomal inner matrix (Akutsu & Jimbow, 1988).

The HMSA-5 antigen showed homology with the antigen recognised by MoAb TA99 as tested by sequential immunodepletion and by partial peptide mapping of the immunoprecipitated proteins. MoAb TA99 identifies an antigen gp75 (Thomson *et al.*, 1985; Tai *et al.*, 1985) 'pigmentation-associated-antigen' (PAA), which was originally described as an antigen immunoprecipitated by serum from a melanoma patient (Mattes *et al.*, 1983). PAA was shown to be present in both melanocytes and melanoma cells (Tai *et al.*, 1983; Houghton *et al.*, 1987). In addition, an inverse correlation between expression of the TA99 antigen and the stage of progression of melanoma tumours was observed (Houghton *et al.*, 1987). Its glycosylation characteristics suggested that this antigen is processed in the trans-Golgi cisternae prior to its localisation on melanosomes (Roux & Lloyd, 1986; Vijayasaradhi & Houghton, 1991).

Immunoelectron microscopy using MoAb TA99 demonstrated a peripheral pattern of reactivity with the outer membrane of mature melanosomes, and little or no reactivity with other organelles (Thomson *et al.*, 1988). MoAb HMSA-5, like MoAb TA99, showed strong ractivity with the cytoplasmic face of the melanosome, but in addition appeared to react positively with small associated uncoated vesicles and regions of the Golgi complex from which these vesicles presumably originate.

A recent study using MoAb TA99 has shown very similar results to those described here with regard to some aspects of the biosynthesis, turnover and glycosylation of gp75 (Vijayasaradhi *et al.*, 1990). However, these authors consistently detect two discrete forms of the mature gp75 molecule with MoAb TA99. They propose the existence of alternatively glycosylated mature forms of the molecule, or alternatively spliced transcripts of the gene leading to two different polypeptides, to explain their results. In contrast, MoAb HMSA-5 appears to immunoprecipitate only one mature form of gp75.

Researchers using another antibody, MoAb 2G10, which has reactivity against gp75 (Cuomo *et al.*, 1991) have recently described their detection of plasma membrane localised gp75 by surface iodination and proteolysis studies (Giacomini *et al.*, 1992). Their results is in strong agreement with our vectorial iodination data which showed modest amounts of HMSA-5 precipitable gp75 being labelled on the cell surface.

Researchers attempting to clone the gene for murine tyrosinase by immunological screening of cDNA expression libraries (Jackson, 1988) or by differential hybridisation between murine melanoma and neuroblastoma cDNA libraries (Shibahara *et al.*, 1986), isolated several clones which did not map to the *c*-locus (*color*; tyrosinase) on mouse chromosome 7 as expected (Jackson, 1988; Kwon *et al.*, 1987*b*; Kwon *et al.*, 1989). Instead, these clones mapped at the b (*brown*) locus on mouse chromosome 4 and encoded a protein which had limited homology (40–50%) with murine tyrosinase (Jackson, 1988; Shibahara *et al.*, 1986; Kwon *et al.*, 1989). Peptide sequencing of affinity-purified human TA99 antigen showed 90% identity with regions of the deduced amino acid sequence of one of these murine clones pMT4 (Vijayasaradhi *et al.*, 1990). When oligonucleotide probes based on this gp75 amino acid sequence information were used to screen a human melanoma cDNA library, a clone termed GP 75-1 was isolated. It is believed that this clone encodes the human counterpart of the murine *b*-locus gene product (Vijayasarshi *et al.*,1990). At the same time, the full nucleotide sequence encoding human tyrosinase-related protein (TRP) was reported and is believed to be identical to the b-locus gene (Cohen *et al.*, 1990).

The mouse b locus gene mapped to mouse chromosome 4 and its genomic organisation has been analysed (Shibahara *et al.*, 1991) while the human homologue, termed CAS2, has been mapped to human chromosome 9p22-pter (Chintamaneni *et al.*, 1991). Other workers have demonstrated that the gp75/b-locus/TRP protein has a measurable catalase activity and have speculated on the role for such an enzyme in controlling pigment production in mammals (Halaban & Moellmann, 1990). Alternative suggestions for the functional identity of the b-locus gene product are two other enzymes involved in post-tyrosinase regulation of melanogenesis; namely 5,6 dihydroxyindole conversion factor and dopachrome oxidoreductase (Muller *et al.*, 1988; Jackson, 1988). Although it does appear to play a regulatory role in melanogenesis as shown by retroviral infection with the murine b locus gene (Bennett *et al.*, 1990), its exact function remains to be elucidated.

During the characterisation of MoAb HMSA-5 described in this report, we found that it may recognise the same glycoprotein as MoAb TA99 and could therefore be a useful reagent for analysing gp75 expression. MoAb HMSA-5 reacts with some melanoma cell lines (including G361) which are amelanotic under our culture conditions, whereas MoAb TA99 is reportedly only reactive with melanised, differentiated melanoma cells (Vijayasardhi & Houghton, 1991). In addition, MoAb TA99 was reported to react weakly with keratinocytes adjacent to melanocytes in both fetal and adult skin (Thomson *et al.*, 1985) whereas MoAb HMSA-5 reactivity is restricted to the melanocytes. Both of these observations may be due to differences in the epitopes recognised by the two antibodies on a common target glycoprotein. In the latter observation, it may be that the TA99 epitope is partially retained after transfer of melanosomes to keratinocytes, whereas the HMSA-5 epitope is masked or lost. If so, the two antibodies might ideally be used in conjunction with one another to study the *b*-locus gene product expression and distribution in both normal and neoplastic melanocytes.

The authors would like to acknowledge the technical assistance of Mrs H. Marusyk, Mrs Bozena Widtman, Mr Hua Chen and Mrs Linda Gluth. The authors wish to thank Drs Doug Green and Vern

Paetkau for helpful discussion during the course of this work. The authors acknowledge the generous support of the Medical Research Council of Canada and the National Cancer Institute of Canada. J.E. Der was the recipient of an MRC postdoctoral fellowship during the tenure of this work. T. Horikoshi was a visiting scholar to the Division of Dermatology and Cutaneous Sciences at the University of Alberta.

### References

AKUTSU, Y. & JIMBOW, K. (1986). Development and characterization of a mouse monoclonal antibody, MoAb HMSA-1, against a melanosomal fraction of human malignant melanoma. *Cancer Res.*, **46**, 2904–2911.

AKUTSU, Y. & JIMBOW, K. (1988). Immunoelectron microscopic demonstration of human melanosome associated antigens (HMSA) on melanoma cells: comparison with tyrosinase distribution. *J. Invest. Dermatol.*, **90**, 179–184.

BALE, S.J., DRACOPOLI, N.C., TUCKER, M.A., CLARK, W.H., FRASER, M.C., STANGER, B.Z., GREEN, P., DONIS-KELLER, H., HOUSMAN, D.E. & GREENE, M.H. (1989). Mapping the gene for hereditary cutaneous malignant melanoma-dysplastic nevus to chromosome 1p. *New Eng. J. Med.*, **320**, 1367–1371.

BENNETT, D.D., HUSZAR, D., LARPIS, P.J., JAENISCH, R. & JACKSON, I.J. (1991). Phenotypic rescue of mutant brown melanocytes by a retrovirus carrying a wild-type tyrosinase-related protein gene. *Development*, **110**, 471–475.

CHINTAMANENI, C.D., RAMSAY, M., COLMAN, M.-A., FOX, M.F., PICKARD, R.T. & KWON, B.S. (1991). Mapping the human Cas 2 gene, the homologue of the mouse brown (b) locus, to human chromosome 9p22-pter. *Biochem. Biophys. Res. Commun.*, **178**, 227–235.

COHEN, T., MULLER, R.M., TOMITA, Y. & SHIBAHARA, S. (1990). Nucleotide sequence of the cDNA encoding human tyrosinase-related protein. *Nucleic Acids Res.*, **18**, 2807–2808.

CUOMO, M., NICOTRA, M.R., APOLLONJ, C., FRAIOLI, R., GIACOMINI, P. & NATALI, P.G. (1991). Production and characterization of the murine monoclonal antibody 2G10 to a human T4-tyrosinase epitope. *J. Invest. Dermatol.*, **96**, 446–451.

EAGER, K.B. & KENNET, R.H. (1983). The use of conventional antisera in the production of specific monoclonal antibodies. *J. Immunol. Methods*, **64**, 157.

EISINGER, M. & MARKO, O. (1982). Selective proliferation of normal human melanocytes *in vitro* in the presence of phorbol ester and cholera toxin. *Proc. Natl Acad. Sci. USA*, **79**, 2018–2022.

ELBEIN, A.D. (1987). Inhibitors of the biosynthesis and processing of N-linked oligosaccharide chains. *Annual Rev. Biochem.*, **56**, 497–534.

GARCIA, P.D., OU, T.H., RUTTER, W.T. & WALTER, P. (1988). Targetting of the Hepatitis B Virus precore protein to the endoplasmic reticulum membrane: after signal peptide cleavage translocation can be aborted and the product released into the cytoplasm. *J. Cell Biol.*, **106**, 1093–1104.

GIACOMINI, P., FRAIOLI, R., CUOMO, M. & NATALI, P.G. (1992). Membrane compartmentalization of melanosomal gp75. *J. Invest. Dermatol.*, **98**, 340–342.

GRIFFITHS, G., QUINN, P. & WARREN, G. (1983). Dissection of the Golgi complex. I. Monensin inhibits the transport of viral membrane proteins from medial to trans Golgi cisternae in baby hamster kidney cells infected with Semliki forest virus. *J. cell Biol.*, **96**, 835–850.

HALABAN, R. & MOELIMANN, G. (1990). Murine and human b locus pigmentation genes encode a glycoprotein (gp75) with catalase activity. *Proc. Natl Acad. Sci. USA*, **87**, 4809–4813.

HARLOW, E. & LANE, D. (1988). Partial proteolytic peptide maps -V8 protease. In: *Antibodies, A Laboratory Manual.* Harlow, E & Lane, D., (eds) pp. 641–642, Cold Spring Harbor Laboratory Press: N.Y.

HAYASHIBE, K., MISHIMA, Y., ICHIHASHI, M & KAWAI, M. (1986). Melanosomal antigenic expression on the cell surface and intracellular subunits within melanogenic compartments of pigment cells: analysis by antimelanosome-associated monoclonal antibody. *J. Invest. Dermatol.*, **87**, 89–94.

HEARING, V.J. & JIMENEZ, M. (1989). Analysis of mammalian pigmentation at the molecular level. *Pigment Cell Res.*, **2**, 75–85.

HERLYN, M., CLARK, W.H., RODECK, U., MANCIANTI, M.L., JAMBROSIC, J. & KOPROWSKI, H. (1987). Biology of tumor progression in human melanocytes. *Lab. Invest.*, **56**, 461–474.

HERLYN, M., THURIN, J., BALABAN, G., BENNICELLI, J.L., HERLYN, D., ELDER, D.E., BONDI, E., GUERRY, D., NOWELL, P., CLARK, W.H. & KOPROWSKI, H. (1985). Characteristics of cultured human melanocytes isolated from different stages of tumor progression. *Cancer Res.*, **45**, 5670–5676.

HOUGHTON, A.N., REAL, F.X., DAVIS, L.J., CORDON-CARDO, C. & OLD, L.J. (1987). Phenotypic heterogeneity of melanoma. Relation to the differentiation program of melanoma cells. *J. Exp. Med.*, **165**, 812–829.

IMOKAWA, G. (1990). Analysis of carbohydrate properties essential for melanogenesis in tyrosinases of cultured malignant melanoma cells; by differential carbohydrate processing inhibition. *J. Invest. Dermatol.*, **95**, 39–49.

JACKSON, I.J. (1988). A cDNA encoding tyrosinase-related protein maps to the brown locus in mouse. *Proc. Natl acad. Sci. USA*, **85**, 4392–4396.

JIMBOW, K. (1986). Biology of melanocytes: cellular and subcellular control of melanocyte differentiation. In *Brown Melanoderma: Biology and Disease of Epidermal Pigmentation.* Fitzpatrick, T.B., Wick, M. & Toda, K., (eds), pp. 45–61. University of Tokyo Press: Tokyo.

JIMBOW, K. & FITZPATRICK, T.B. (1973). Characterization of a new melanosomal structural component – the vesiculoglobular body – by conventional transmission, high voltage, and scanning electron microscopy. *J. Ultrastruct. Res.*, **48**, 269–283.

JIMBOW, K., YAMANA, K., AKUTSU, Y. & MAEDA, K. (1988). Nature and biosynthesis of structural matrix proteins in melanosomes: melanosomal structural protein as differentiatin antigens for neoplastic melanocytes. In Advances in pigment cell research. In *Progress in Clinical and Biological Research*, **256**, pp. 169–182. Alan R. Liss: N.Y.

JIMBOW, K., JIMBOW, M. & PAETKAU, V. (1990). Analysis of human melanosome-specific antigen in malignant melanoma and its precursors, In *Human Melanoma: From Basic Research to Clinical Application*, Ferrone, S. (ed), pp. 31–50. Springer-Verlag: New York.

KWON, B.S., HALABAN, R., KIM, G.S., USACK, L., POMERANTZ, S. & HAQ, A.S. (1987*a*). A melanocyte-specific complementary DNA clone whose expression is inducible by melanotropin and isobutylmethyl xanthine. *Mol. Biol. Med.*, **4**, 339–355.

KWON, B.S., HAQ, A.K., POMERANTZ, S.H & HALABAN, R. (1987*b*). Isolation and sequence of a cDNA clone for human tyrosinase that maps at the mouse c-albino locus. *Proc. Natl Acad. Sci. USA*, **84**, 7473–7477.

KWON, B.S., HAQ, A.K., WAKULCHIK, M., KESTLER, D., BARTON, D.E., FRANKE, V., LAMOREUX, L., WHITNEY, J.B. & HALABAN, R. (1989). Isolation, chromosomal mapping, and expression of the mouse tyrosinase gene. *J. Invest. Dermatol.*, **93**, 589–594.

LAEMMLI, U.K. (1970). Cleavage of structural proteins during the assembly of the head of bacteriophage T4. *Nature*, **227**, 665–666.

LASKEY, R.A. & MILLS, A.D. (1975). Quantitative film detection of $^{3}H$ and $^{14}C$ in polyacrylamide gels by fluorography. *Europ. J. Biochem.*, **56**, 334–341.

LEWIS, V., GREEN, S.A., MARSH, M., VIHKO, P., HELENIUS, A. & MELLMAN, S. (1985). Glycoproteins of the lysosomal membrane. *J. Cell Biol.*, **100**, 1839–1847.

LIPPINCOTT-SCHWARTZ, J., YUAN, L.C., BONIFACINO, J.S. & KLAUSNER, R.D. (1989). Rapid redistribution of golgi proteins into the ER in cells treated with brefeldin A: evidence for membrane cycling from golgi to ER. *cell*, **56**, 801–813.

MAEDA, K. & JIMBOW, K. (1988). Specification and use of a mouse monoclonal antibody raised against melanosomes for the histopathologic diagnosis of amelanotic malignant melanoma. *Cancer*, **62**, 926–934.

MATTES, M.J., THOMSON, T.M., OLD, L.J. & LLOYD, K.O. (1983). A pigmentation-associated, differentation antigen of human melanoma defined by a precipitating antibody in human serum. *Int. J. Cancer*, **32**, 717–721.

MCEWAN, M., PARSONS, P.G., MOSS, D.J., BURROWS, S., STENZEL, D., BISHOP, C.J. & STRUTTON, G.M. (1989). Monoclonal antibody against a melanosomal protein in melanotic and amelanotic human melanoma cells. *Pigment Cell Res.*, **2**, 1–7.

MULLER, G., RUPPERT, S., SCHMID, E. & SCHUTZ, G. (1988). Functional analysis of alternatively spliced tyrosinase gene transcripts. *EMBO J.*, **7**, 2723–2730.

NOWELL, P.C. (1989). Chromosomal and molecular clues to tumor progression. *Seminars in Oncol.*, **16**, 116–127.

OIKAWA, A., SAEKI, H., AKIYAMA, T. & MATSUMOTO, J. (1987). Electron microscopic evidence for stimualtion of melanosomal maturation by lysosomotropic agents and monensin in cultured B16 mouse melanoma cells. *Pigment Cell Res.*, **1**, 44–50.

ROUX, L. & LLOYD, K.O. (1986). Glycosylation characteristics of pigmentation-associated antigen (GP 75): an intracellular glycoprotein of human melanocytes and malignant melanomas. *Arch. Biochem. Biophys.*, **251**, 87–96.

SHIBAHARA, S., TAGUCHI, H., MULLER, R.M., SHIBATA, K., COHEN, T., TOMITA, Y. & TAGAMI, H. (1991). Structural organization of the pigment cell-specific gene located at the brown locus in mouse: its promoter activity and alternatively spliced transcripts. *J. Biol. Chem.*, **266**, 15895–15901.

SHIBAHARA, S., TOMITA, Y., SAKAKURA, T., NAGER, C., CHAUDHURI, B. & MULLER, R. (1986). Cloning and expression of cDNA encoding mouse tyrosinase. *Nucleic Acids Res.*, **14**, 2413–2427.

TAI, T., EISINGER, M., OGATA, S.-I. & LLOYD, K.O. (1983). Glycoproteins as differentiation markers in human malignant melanoma and melanocytes. *Cancer Res.*, **43**, 2773–2779.

TAKAHASHI, H., HORIKOSHI, T. & JIMBOW, K. (1985). Fine structural characterization of melanosomes in dysplastic nevi. *Cancer*, **56**, 111–123.

THOMSON, T.M., MATTES, M.J., ROUX, L., OLD, L.J. & LLOYD, K.O. (1985). Pigmentation-associated glycoprotein of human melanomas and melanocytes: definition with a mouse monoclonal antibody. *J. Invest. Dermatol.*, **85**, 169–174.

THOMSON, T.M., REAL, F.X., MURAKAMI, S., CORDON-CARDO, C., OLD, L.J. & HOUGHTON, A.N. (1988). Differentiation antigens of melanocytes and melanoma: analysis of melanosome and cell surface markers of human pigmented cells with monoclonal antibodies. *J. Invest. Dermatol.*, **90**, 459–466.

VIJAYASARADHI, S., BOUCHARD,B. & HOUGHTON, A.N. (1990). The melanoma antigen gp75 is the human homologue of the mouse b (BROWN) locus gene product. *J. Exp. Med.*, **171**, 1375–1380.

VIJAYASARADHI, S. & HOUGHTON, A.N. (1991). Purification of an autoantigenic 75-kDa human melanosomal glycoprotein. *Int. J. Cancer*, **47**, 298–303.